# How people with chronic obstructive pulmonary disease perceive their illness: a qualitative study between mind and body

**DOI:** 10.1186/s12890-020-1157-3

**Published:** 2020-05-04

**Authors:** Marta Pozzar, Eleonora Volpato, Chiara Valota, Francesco Pagnini, Paolo Innocente Banfi

**Affiliations:** 1 Department of Psychology, Università Cattolica del Sacro Cuore, Milan, Italy; 2 IRCCS Fondazione Don Carlo Gnocchi, Milan, 20148 Italy; 3 Department of Psychology, Harvard University, Cambridge, MA USA

**Keywords:** Illness perception, Chronic obstructive pulmonary disease, Mind/body connection, Qualitative research, Patient’s experience

## Abstract

**Background:**

Although many studies on people with Chronic Obstructive Pulmonary Disease (COPD) have examined the mutual impact of physical status and emotional experience, there is limited knowledge about the way COPD people first-hand perceive their condition. This study was designed to investigate the illness perceptions of the patients and, secondarily, to explore their beliefs about the mind-body relationship.

**Methods:**

This qualitative study has exploited an ad-hoc semi-structured interview to collect personal perspectives of participants on their illness. Twenty-seven patients (15 males and 12 females), with a mild to severe COPD, were recruited within the Respiratory Rehabilitation Unit of Don Carlo Gnocchi Foundation, in Milan. The thematic analysis of the interviews’ content was facilitated by NVivo (12th version, QSR International®).

**Results:**

The thematic analysis of the corpus resulted in four master themes. *Illness experience* has been considered the primary one. Indeed, dealing with COPD every day allows these people to portray a specific representation of the *mind-body relationship,* to gain a certain degree of *expertise* and to develop a perspective on the *future*.

**Conclusions:**

Individual perceptions of the illness vary among people with COPD, but some common experiences characterize them. Many patients share a profound belief that their mental state and their physical symptoms are highly interrelated.

## Background

Chronic Obstructive Pulmonary Disease (COPD) is a common, preventable and treatable disease that is characterized by persistent respiratory symptoms and airflow limitation that is due to airway and/or alveolar abnormalities usually caused by significant exposure to noxious particles or gases [1].

Together with the physical symptoms, the illness may have a strong psychological impact on the lives of the patients. Anxiety and depression are common in this population, with a prevalence of 10–55% [2] and 25% [3] respectively. Several aspects (i.e., loss of autonomy, uncertainties about the future) may stimulate or extend depressive symptoms [4, 5]. Similarly, other symptoms (e.g., the inability to breathe properly) may trigger anxious reactions [6, 7].

While physical symptoms influence the psychological domains, the reverse is also true: for example, depression and anxiety seem to increase the risk of mortality [8], to raise the number of hospitalizations due to exacerbations [9], to increment dyspnoea episodes [10], and to worsen patients’ health-related quality of life [11]. Anxiety and depression are not the only psychological aspects that could impact the physical well-being: emotional and cognitive components of the illness perception can influence behavioural patterns, such as treatment adherence and exercise [12], and expectations have been found to be very important in the progression of many diseases [13]. These are patterns that have been explored by several studies [14] and are therefore well known by many researchers. Some reviews have focused their attention on the illness perception, for example condensing the most relevant findings on the unmet needs of COPD patients (i.e., physical, psychosocial, informational and professional care needs) [15], or taking into account the impact of the disease on patients’ lives (i.e., existential and psychosocial longings, loss of hope and meaning, symptoms burden) [16]. As far as we know, however, no study has specifically explored the COPD patients’ beliefs about this mind/body relationship.

Despite a relatively large amount of quantitative studies, there are comparatively few qualitative studies that have explored the perspectives of patients with respiratory conditions [17]. However, it could hide a concrete potential, since patients’ perspectives may suggest improved interventions and help to understand the patients’ needs [18]. It is possible to find some recurring focuses adopted by researchers: some of them investigated the emotional burden associated with the illness [19–21], especially in acute circumstances like exacerbations [22]; other works explored the process of adaptation following the diagnosis [23, 24] and the daily struggle determined by the symptoms [25]; other studies considered the different experiences lived by male and female patients [26].

Therefore, the rationale of the study was to expand earlier qualitative research on the patient’s perceived relationship between illness, mind and body for identifying cognitive and emotional representations. This knowledge may help to improve health care communication in COPD and optimize both adherence and quality of life.

Given the paucity of contributions that directly examine the patients’ perspective, we designed a qualitative study that directly explores the illness perceptions of COPD people. Moreover, we aimed to collect novel information about their beliefs about the relationship that the mind and the body can have in their illness experience.

## Methods

### Study design

We carried out a qualitative study of individual face to face semi-structured interviews (Table 1) analyzed by thematic analysis [27, 28].

**Table 1:** Semi-structured interview schedule

• Based on your experience, what are the most disabling symptoms that you must deal within your daily life? • What kind of emotions originated from these experiences over time? • Many patients feel “anxious” and/or “depressed”. How do you imagine these emotions may affect the physical course of the disease? • Starting from your previous considerations, which kind of relationship do you think may exist between emotions and COPD typical symptoms? • How do you expect your clinical framework may evolve in the future? • Would you like to add any other consideration?	

We identified the relevant domains, inspired by our clinical experience**.** We then translated the domains into more detailed categories, then transformed into open-ended questions, useful to promote articulated answers. The choice of semi-structured form had the purpose of ensuring both flexibility and structure during the data collection and data analysis processes.

### Participants

**Fig. 1:**
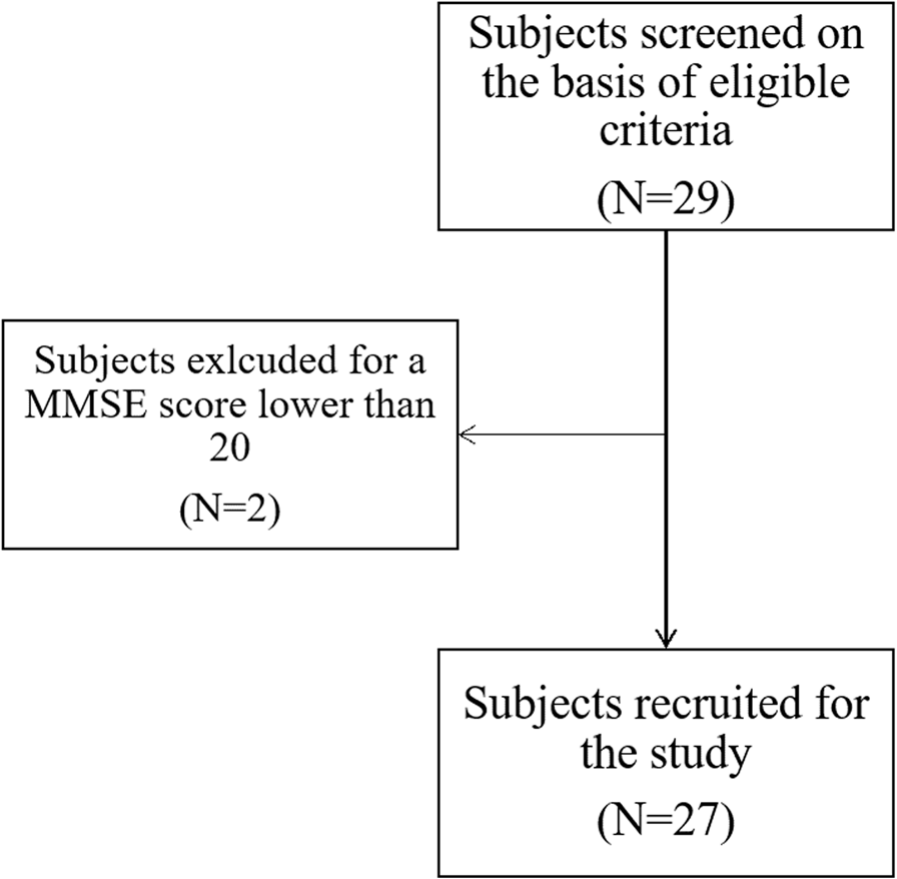
Participants’ recruitment. A flow-chart showing the recruitment process

Participants were referred to the rehabilitation program by a pulmonologist, who invited them to take part in the study. They were included if they had a spirometry-confirmed COPD diagnosis, from a mild (FEV_1_^1^≥80% predicted) to a very severe stage (FEV_1_ < 30% predicted) according to the Global Initiative for Obstructive Lung Disease (GOLD) criteria [1], if they agreed to join the study and they signed the Consent Form, and if they were over 18 years of age. People with cognitive impairment (MMSE^2^ lower than 20) or another serious medical condition were excluded (Fig. 1).

We opted for a theoretical sampling, interrupting the recruitment when the theoretical saturation was reached [29]. In this regard, concerning the fairly narrow aim of the study, the degree of structure of the interviews and the relatively homogenous population, the analysis was conducted after every 6 interviews and data saturation was determined when no new codes, themes or patterns emerged [30]. Participants were recruited inside the Respiratory Rehabilitation Unit of Don Carlo Gnocchi Foundation, in Milan. Twenty-seven participants were included in the study.

Participants were informed in advance about the central topics of the conversation, the principles concerning voluntary participation, anonymity and their right to withdraw from the study at any time. They all provided informed consent to the study participation. The study was conducted following the principles of the Helsinki Declaration and approved by the Don Gnocchi Foundation Ethical Committee.

### Data collection

Data were collected between February and June 2018. All the interviews were conducted inside the Unit, in a dedicated room, by a psychologist never met before by the participants. When not doable, participants were interviewed in their rooms, trying to keep the setting as quiet and undisturbed as possible. Each interview lasted, on average, between 10 and 15 min and was audio-recorded under the explicit consent of the patient. Before starting, the researcher used to remind the participants to feel free to answer with the level of detail they preferred. Moreover, they were invited to express freely, paying little attention to grammar, as the focus was all on their personal experience and thoughts. The researcher, additionally, exploited some prompts to help the participant express his/her point of view and to deepen the topics of major interest.

### Data analysis

Audio-recorded interviews were transcribed verbatim. A thematic analysis of the content was conducted through NVivo (12th version, QSR International®), by two researchers (E.V. and M.P.). The initial coding of all transcripts was carried out by the two researchers. The analysis followed the six stages described by Braun and Clarke [27]: familiarization with data, generating initial codes, searching for themes, reviewing themes, defining and naming themes, and producing the report. A third researcher (C.V.) considered the subgroups in relation to the severity of the disease and all researchers discussed to ensure agreement on the emerged themes. A reflexivity log was kept during all the analyses to allow that themes remained grounded with the data set [28]. Moreover, the analyses confirmed that the data saturation was achieved and no findings after 27 interviews.

Also, a semantic analysis was conducted. Some specific aspects were considered, i.e. the most common emotions, the most used verbs (both with a positive and a negative meaning) and the most meaningful keywords occurring in the text. Finally, some word-clouds were generated using NVivo software to visualize the focus on the most frequent words and represent graphically the previously described features.

## Results

The characteristics of the participants are summarized in Tables 2 and 3.

**Table 2:** Participants’ information

	*N*	Mean (*SD*)
Gender		
Male	15	
Female	12	
Age		72.37 (±9.44)
Educational level		
None	1	
Primary school	9	
Lower secondary school	7	
Upper secondary school	7	
Higher education	4	
NIV users	12	
LTOT users	21	
Ex-smokers	21	
FEV_1_ in % predicted		41.58 (±23.45)
GOLD 1	1	122 (±0)
GOLD 2	5	64 (±11.47)
GOLD 3	9	42.88 (±4.34)
GOLD 4	11	23 (±5.31)
Total	27	

*NIV* Non-invasive Ventilation, *LTOT* Long-term Oxygen Therapy, *FEV*_*1*_ Forced Expiratory Volume in 1 second, *N* Numerosity, *SD* Standard Deviation

**Table 3:** Participants’ demographic characteristics in detail

ID	Gender	Age	LTOT	NIV	Smoker	Years of smoking	Fev_1_ (% of predicted)	COPD severity (GOLD)
1	Male	1	Yes	No	Yes	32	11	Very severe
2	Male	3	Yes	Yes	Ex-smoker	40	23	Very severe
3	Female	2	Yes	Yes	Ex-smoker	46	22	Very severe
4	Male	3	Yes	No	Ex-smoker	57	48	Severe
5	Male	3	Yes	Yes	Ex-smoker	30	76	Moderate
6	Female	2	No	No	No	0	56	Moderate
7	Male	1	No	Yes	Ex-smoker	25	Unknown	Unknown
8	Female	1	Yes	No	Ex-smoker	36	29	Very severe
9	Female	3	Yes	Yes	Ex-smoker	50	57	Moderate
10	Female	2	Yes	Yes	No	0	19	Very severe
11	Male	1	Yes	No	Ex-smoker	42	21	Very severe
12	Female	2	Yes	No	Ex-smoker	56	27	Very severe
13	Male	3	Yes	No	Ex-smoker	65	40	Severe
14	Male	3	No	No	No	0	122	Mild
15	Male	2	No	No	Ex-smoker	45	39	Severe
16	Female	2	Yes	No	Ex-smoker	50	27	Very severe
17	Male	1	Yes	Yes	Ex-smoker	38	36	Severe
18	Male	2	Yes	No	Ex-smoker	25	25	Very severe
19	Male	3	Yes	Yes	Ex-smoker	15	29	Very severe
20	Female	3	Yes	No	Ex-smoker	44	54	Moderate
21	Female	2	Yes	Yes	Ex-smoker	50	48	Severe
22	Male	3	Yes	No	Ex-smoker	40	46	Severe
23	Female	3	No	Yes	No	0	77	Moderate
24	Female	3	Yes	No	Ex-smoker	10	46	Severe
25	Male	3	Yes	No	Ex-smoker	57	20	Very severe
26	Male	3	Yes	Yes	Ex-smoker	48	40	Severe
27	Female	3	No	Yes	No	0	43	Severe

ID, Identifier (of the participant); For anonymity reasons, the Age of participants is provided as age-ranges [1, (51-64 years)] [2, (65-74 years)] [3, (75-86 years)]

**Fig. 2:**
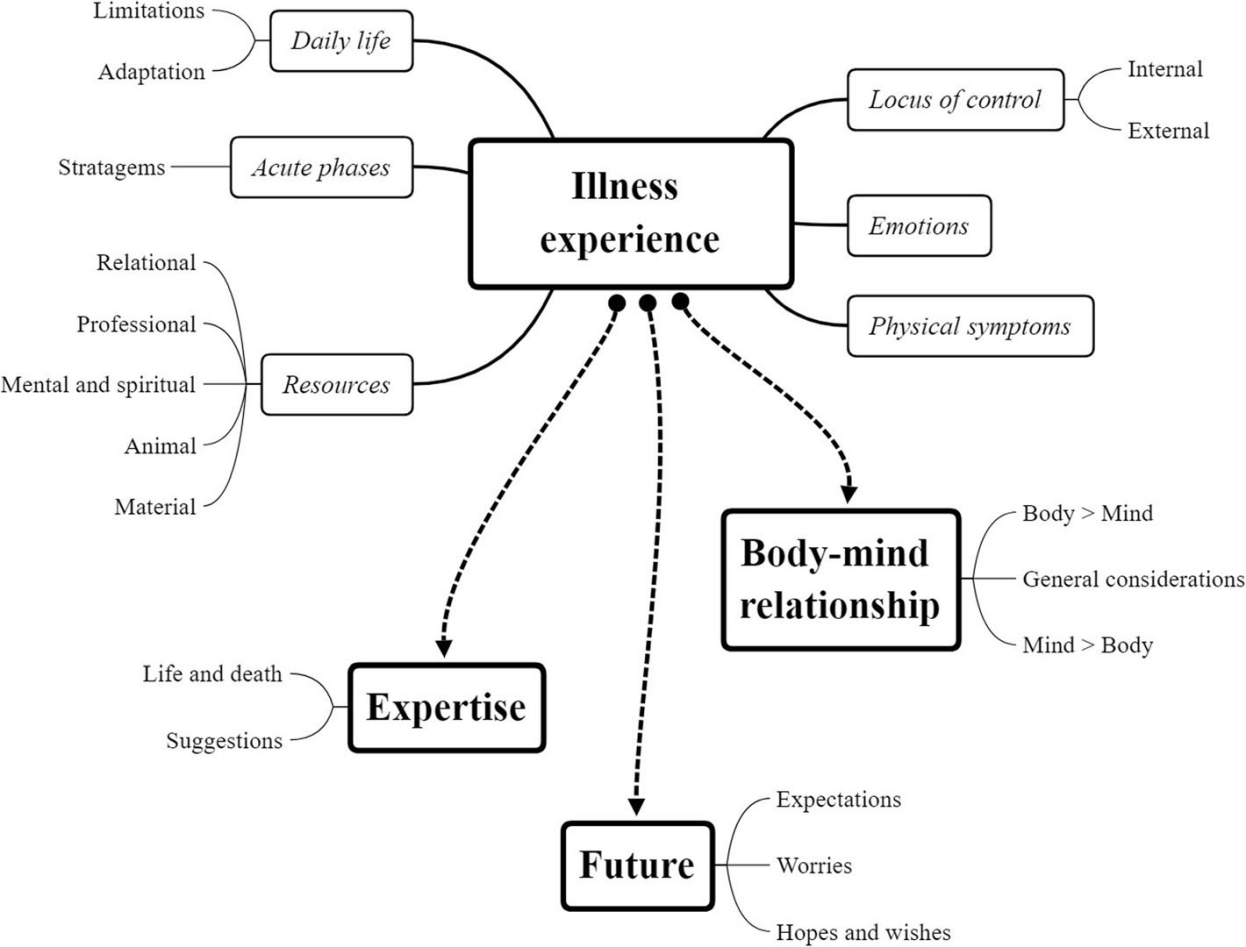
Themes map. A map showing the themes emerged from the thematic analysis and their connection

The thematic analysis resulted in four master themes: “illness experience”, “mind-body relationship”, “expertise”, and “future”. These themes and their connection are shown in Fig. 2.

### Illness experience

All the participants point out aspects concerning their daily life. A recurring concept is that of *limitation*. This word can be thought both as a concrete obstacle in the execution of everyday activities and hobbies.

‘I just can’t do what I want. I feel exhausted.’ [ID:5]; ‘Each move causes me strain and each move takes my breath away’ [ID:8]; ‘Even sitting in my bedroom. I cannot go to bed by myself, someone must help me. To dress up, the same. To take off my clothes, the same’ [ID:10]; ‘What I miss the most is my job’ [ID:17],

and, at the same time, as a mental limit forcing the person to live in the fear that the worst might happen:

‘The point is: I want to go out, but I can’t.’ [ID:23]; ‘What if I go out to buy the bread and suddenly have a crisis? What would I do?’ [ID:8]; ‘If there’s something that bothers me, it’s when I have a coughing fit with too much mucus in public … it’s irrepressible and repulsive and you’re never really ready.’ [ID:2].

Another aspect that emerged from the words of participants is the struggle in *accepting* the situation, not only for the personal implications but also for the burden they are afraid to put on the others.

‘Everything that represents a limit bothers me’ [ID:7]; ‘And then you feel like an inconvenience for the others [ …], you feel useless’ [ID:8]; ‘ … it prevents me from being self-sufficient … when they have to change my diaper, I feel -somehow- embarrassed. I want to achieve autonomy, being able to complete these actions by myself’ [ID:3]; ‘I feel like a burden for my husband. He is ten years older than me, he’s 80. I feel sorry because he has to replace me for everything’ [ID:12].

The *adaptation* process determines highs and lows, shifting from an extreme effort in coping with the disease to severe moments of discouragements:

‘I’m a little bit upset from this point of view … I mean, you are missing out on something, of your personality, of your lifestyle, of your attitudes and behaviours … ’ [ID:25]; ‘If something goes wrong, it doesn’t matter, you must try to go on’ [ID:3]; ‘You get to the point you feel like you are on your last leg. It is hard to accept it, it is hard to live with it, it changes way too much your normal life’ [ID:8]; ‘Sometimes I feel discouraged for not being able to do what I’d like to do … there are peaks and valleys. Now I feel a little bit better.’ [ID:6].

The experience of the pathology concerns also the *acute phases*, able to trigger terrible feelings:

‘When you cannot breathe, it’s panic. Total panic. [ …] It feels like a plastic bag on your face. Maybe it lasts a few seconds, but you feel like dying’ [ID:8] ‘When I can’t breathe, it’s awful … you feel like suffocating.’ [ID:10]; ‘To me, the lack of breath is something worse than the pain’ [ID:19].

Everyone chooses *different strategies* to answer these dramatic events: drinking water, keeping calm or practicing breathing exercises. Anyway, the *locus of control* plays a fundamental role in modulating the reaction. Some people believe there is an external cause for their illness:

‘All of the doctors gave me so many medicines they made me ill’ [ID:15]; ‘I’ve caught a cold here: here and at the gym’ [ID:1]; ‘I don’t know how this happened’ … ‘why so all of a sudden?’ [ID:13].

Other participants, differently, can recognize their responsibility:

‘If I only had quitted smoking years ago, maybe now I would have not found myself in this situation’ [ID:12]; ‘I used to have recurring bronchitis, but didn’t follow the treatments with antibiotics … because I was not constant … so I have been dragging it for years and years, plus the smoke and the poor diet … I finally got this problem’ [ID:21].

and, consequently, to actively manage their selves and the pathology:

‘The most important thing is always believing. Having will power and go on. And then you get through anything’ [ID:4]; ‘You should try to be optimist, to react’ [ID:9].

For what concerns smoke, a strong repulsion towards the past habits emerges, together with the hope for a better condition now that cigarettes have been set aside:

‘I hope that having stopped smoking will bring me to see life differently and to recover’ [ID:12].

An element common to all the participants, in different forms and levels of severity, is the experience of crippling *physical symptomatology*. Above all, the dyspnoea represents the most tragic issue:

‘When the little space where the air flows gets obstructed, for me it is like having a blown-up balloon and the air cannot get in! That is the feeling. You try to inhale and feel like suffocating’ [ID:8].

Beside dyspnoea, people lament other symptoms such as back and legs pain, intestinal, cardiac, urinary and musculoskeletal problems, a generic and prolonged sense of tiredness, tremors, phlegm accumulation, and dizziness.

This varied and complex physical profile of the person suffering from COPD reflects an equally multifaceted *emotional experience*. On the *negative* side, we can find feelings of anxiety (discomfort, apprehension, anxiety, and panic), sadness (displeasure, degradation, restlessness, melancholy, sadness, pessimism, and depression), annoyance and nervousness; primary emotions like anger and fear and, lastly, social emotions like shame and guilt. On the *positive* sphere, feelings of lightness, tranquillity, happiness, and optimism make up for the bad ones. Unfortunately, negative emotions are much more prevalent than the positives in the narratives.

Finally, the different *resources* people use to deal with this situation can be specified. Most of all, *relational* ones seem to be particularly helpful. Beside them, we can also find professional, animal, mental, spiritual, and practical resources (like medicines, physiotherapy, social networks, books, movies …):

‘I need to see my grandchildren growing up. I still have a family, young sons, so I like it, I like living’ [ID:21]; ‘The cooperation of nurses, doctors … everyone! It helps so much!’ [ID:2] ‘God gave us so many beautiful things, among them the friendship of animals!’ [ID:15]; ‘Humour is fundamental!’ [ID:15]; ‘I like being alive!’ [ID:11]; ‘My life is precious’ [ID:3];

### Body-mind relationship

Two different approaches have been adopted for the question related to the body-mind relationship. Some participants claim that the physical status determines emotions and thoughts, and not the reverse:

‘When that breathing problem arrives, I become 100% anxious’ [ID:26]; ‘Now that I am no longer self-sufficient, it is an unbearable feeling … and the mind works consequently’ [ID:8].

On the other hand, some have an opposing view. It is the mental condition to influence the body:

‘When I was nervous, my breath lacked far more than normal’ [ID:12]; ‘I think the mind may have some consequences on the body. You loosen up, you keep saying “I’m getting worse, I’m getting worse” and the final result is that you don’t even have the strength to react’ [ID:8]; ‘The mind always rules’ [ID:22].

A little part of the interviewees is convinced that the relationship between mind and body does not exist: they travel on parallel lines. A greater part of them, on the contrary, assumes that this relationship subsists and it’s somehow circular:

‘Everything is linked!’ [ID:10]; ‘The more you lack on one side, the more you are affected on the other’ [ID:25]; ‘When I manage my time in a better way, when I am satisfied with myself, then mind and body are happy, like saturated’ [ID:20].

A certain difficulty in reasoning about these issues emerge. The lack of consciousness about these complex themes prevented the participants from answering with a greater level of detail.

### Expertise

Experiencing COPD first-hand involves the gradual development of a certain degree of expertise towards it. First, this chronic and degenerative illness inevitably brings into play the relationship between *life* and *death*. The references to these existential themes are frequent and space from an optimistic and proactive approach to a more negative one:

‘You must live, day to day’ [ID:25]; ‘It’s not logic, it’s life’ [ID:18]; ‘This is the life, a little bit hard, every day you have to fight against it’ [ID:10]; ‘How can someone live … ’ [ID:19]; ‘Why am I even bothering?’ [ID:23]; ‘I want to make it over’ [ID:26]; ‘If at some point I’ll no longer be able to breathe, I’ll certainly think *what the hell* I am doing in the world’ [ID:17].

Secondly, participants can express some *suggestions* based on the challenges won and those that still have to be faced. These motivational sentences are not only related to illness management, but also to life in general:

‘You must keep yourself mentally young’ [ID:18]; ‘Receiving some more information about the consequences (not only the physical ones) of the pathology would be helpful’ [ID:7]; ‘Family should be provided with more help and care’ [ID:8]; ‘It’s important to keep that mood up’ [ID:20]; ‘To make the mind work, read a good book, or watch an interesting movie!’ [ID:14].

Notwithstanding the different perspectives, the need for mental stratagems to make up for physical limitations is a widespread element.

### Future

For what concerns the future, some negative *expectations* about the body have been highlighted by participants:

‘There is no optimism, you know it only gets worse’ [ID:8]; ‘These things exist and will remain. I have no illusion that I will be better in this sense’ [ID:10].

Cognitive impairment is also imagined:

‘I think they would go in tandem’ [ID:25]; ‘The body gets old, so does the mind’ [ID:16].

Only a few participants see the passing of the years as a positive expedient to get better through therapies over time.

Another aspect is that of *worries*. The main causes of this apprehension are related to the ideas of change and unknown, to the fear of not being able to take care of the own physical condition or to the scare of not receiving adequate support.

Finally, the last category that emerged from the analysis concerns *hopes* and *wishes*. The most desired thing is positive aging: physically, a reasonable recovery; mentally, a certain degree of wisdom and acceptance, as well as good cognitive skills. Talking about relations, most of the participants hope to spend the years to come together with their loved ones, especially watching their grandchildren grow. Finally, a common wish is to come back doing some activities quitted because of the disease: taking pictures, swimming, going for walks. To sum up with the words of a woman:

‘I hope to regain a life worth living’ [ID:12].

**Fig. 3:**
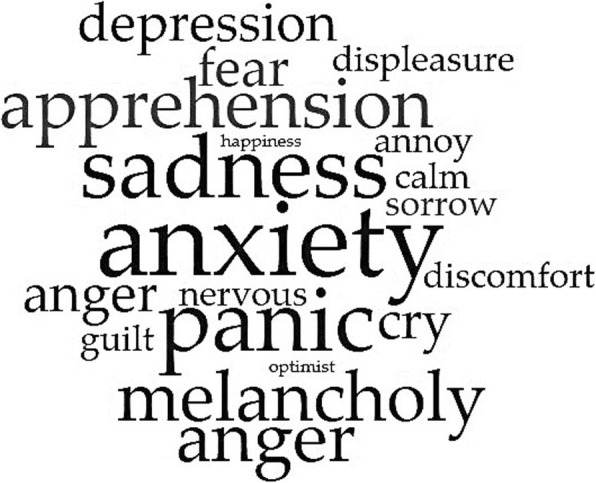
Emotional experience. A word-cloud highlighting the most recurring and meaningful words used by participants to describe their emotional experience

**Fig. 4:**
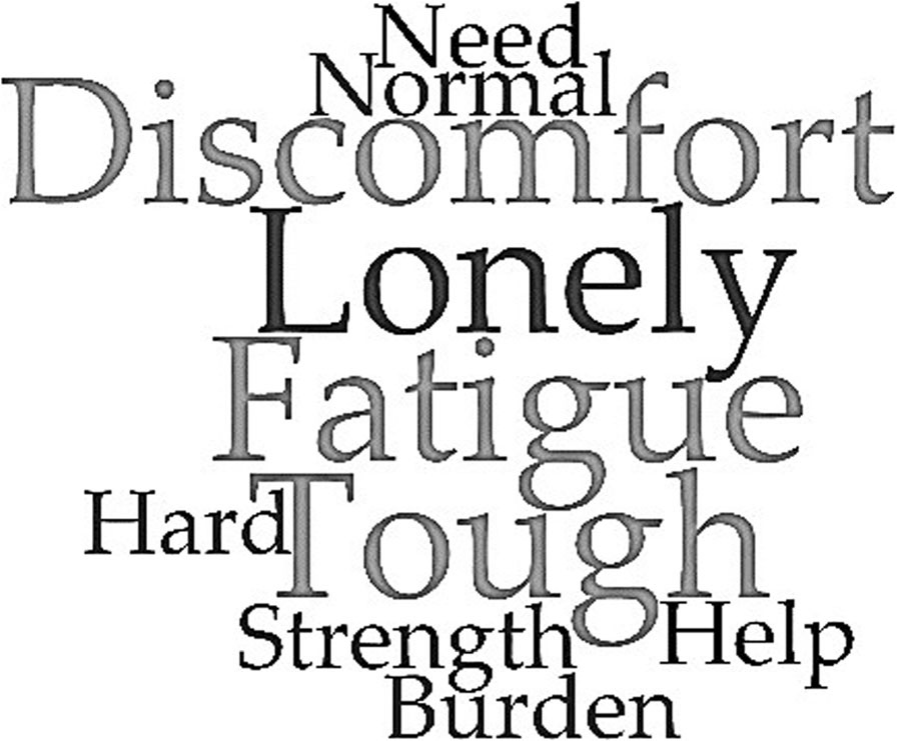
Most used keywords. A world-cloud highlighting the most recurring and meaningful words used by participants to describe their current situation

Finally, the semantic analysis of the corpus resulted in two distinct word-clouds. In the following graphic outputs, words are shown as bigger when their occurrence in the text is higher. Respectively, they represent the emotional experience of participants (Fig. 3) and the ten most used keywords (Fig. 4).

## Discussion

The aim of the study was primarily to understand the way patients perceive their illness and secondarily to catch their representation of the relationship existing between mind and body. The qualitative approach, and the thematic analysis, in particular, allow to identify and interpret patterns of meaning with unique flexibility [27, 28].

The theme of *illness experience* has been logically considered as the origin of the other themes and includes the biggest part of the corpus. Therefore, the daily coexistence with limitations, emergencies, conflicting emotions, and debilitating symptoms qualifies these people as the real experts of COPD. Some of the aspects related to the illness perception have emerged: the different types of locus of control adopted, the emotional response towards the severity of the physical condition and even the appreciation for the medical treatments as a fundamental resource in the adaptation process. This last point, in particular, seems to be consistent with the review conducted by Clari and colleagues, which enhances the pivotal role of healthcare professional care when trying to deal with the disease [15]. Moreover, these findings reflect the connection between illness perceptions, coping, and QoL^3^ that are all associated with each other [31].

Starting from the experience of the pathology, a certain representation of the *body-mind relationship* can be developed. On one hand, it prevails the idea of a monodirectional arrow, linking the body to the mind: the physical condition changes emotions and cognition. On the other hand, it is believed that the mind has the power to rule the body, modulating its ability to work properly and to react. Notwithstanding the contradiction between these two perspectives, the existence of this relationship is not denied, although a certain difficulty in describing it emerges. This was underlined by statements as *‘*I don’t know what to say’ or, in other cases, it could be deducted by the lack of speech. For most of the participants, it probably was the first occasion to debate and reason about this complex issue as well as the first time their lived experience was asked to be put into words.

Another aspect resulting from the direct experience of COPD is the gained *expertise*. It means that patients take on a definite point of view, not only about their condition (that part of illness perception related to “understanding”) but also towards existence itself. Possibly, this effect is emphasized by the specific life-stage crossed by participants, the old age, that easily encourages to draw conclusions about life. In this regard, the last international consensus on COPD stresses the role of self-management interventions to increase the patient’s responsibility for healthcare decisions, considering him/her as the main expert of his/her disease [32].

The last theme, the *future*, brings into play two remaining aspects of illness perception. On one side, the strong concern about what is going to happen. The consciousness about the degenerative process implied by COPD and the consequent certainty of a difficult tomorrow determine a pessimistic and worried perspective. This negative expectation is consistent with what Seamark and colleagues (2004) support with their study: looking forward means dealing with feelings of loss and progressive isolation [33]. On the other side, the main hopes of participants are related to the possibility to preserve their mind active and clear, to keep their mood up and to gain, over time, a deeper acceptance. These aspects will be fundamental to make up for limits and losses, as supported by a meta-synthesis realized by Disler and colleagues [16]. This study, therefore, highlights that keeping positive and taking 1 day at a time can be useful for COPD patients to counteract the feelings of anxiety, the fear of death such as the impression of being, more and more, passive “spectators” of their own lives [16].

The present study includes several limitations. In particular, the advanced age, the low educational level, and the physical fatigue at the time of the interview prevented some of the participants from reasoning in-depth about abstract or excessively complex concepts. Even though we reached theoretical saturation, the small sample does not allow generalization. This population is highly selected, and data was collected in a hospital setting. It is possible that COPD patients reached out in different contexts would provide different answers. Future studies with a bigger and more varied target are required. Moreover, the expertise of the researcher who conducted the interviews might have influenced the participants’ answers, limiting the possibility to consider the other clinical conditions that may impact the quality of life of the patient.

Despite these limits, the study gives a contribution to understanding how people with COPD perceive their illness and depict the relationship between mind and body. Moreover, the possibility to enhance participants’ point of view through an idiographic approach has allowed to point out what living with this pathology means.

The patient’s perspective influences their engagement [34], and therefore their adherence and compliance. The topics that have emerged are therefore relevant from a clinical standpoint. For example, patients for whom the mind/body relationship is particularly relevant, counseling can directly impact the level of adherence [35]. Furthermore, the opportunity of sharing their expertise, or to discuss future perspectives further support the importance of proper psychological support, in particular in group settings.

## Conclusions

Illness perception seems to assume different characteristics from patient to patient, such as the body-mind relationship representation does. What brings together these multiple perspectives, is the idea that a strong bond links physical and mental conditions. This connection, though, is not considered as positive, but rather as a vicious cycle: bad thoughts and feelings cause worse symptomatology and a negative physical status evokes terrible cognitions and emotions.

## Data Availability

The datasets used and/or analyzed during the current study are available from the corresponding author on reasonable request.

## Abbreviations

COPD: Chronic Obstructive Pulmonary Disease
FEV_1_: Forced Expiratory Volume (in 1 s)
GOLD: Global Initiative for Obstructive Lung Disease
ID: Identifier (of the participant)
LTOT: Long-term Oxygen Therapy
MMSE: Mini Mental State Examination
N: Numerosity
NIV: Non-Invasive Ventilation
QoL: Quality of Life
SD: Standard Deviation

## Footnotes

1 FEV_1_: Forced Expiratory Volume in 1 s

2 MMSE: Mini Mental State Examination

3 QoL: Quality of Life
